# Gastroesophageal Reflux Disease and Risk for Bipolar Disorder: A Nationwide Population-Based Study

**DOI:** 10.1371/journal.pone.0107694

**Published:** 2014-09-25

**Authors:** Wan-Shan Lin, Li-Yu Hu, Chia-Jen Liu, Chih-Chao Hsu, Cheng-Che Shen, Yen-Po Wang, Yu-Wen Hu, Chia-Fen Tsai, Chiu-Mei Yeh, Pan-Ming Chen, Tung-Ping Su, Tzeng-Ji Chen, Ti Lu

**Affiliations:** 1 Department of Psychiatry, Kaohsiung Veterans General Hospital, Kaohsiung, Taiwan; 2 Institute of Public Health, National Yang-Ming University, Taipei, Taiwan; 3 School of Medicine, National Yang-Ming University, Taipei, Taiwan; 4 Division of Hematology and Oncology, Department of Medicine, Taipei Veterans General Hospital, Taipei, Taiwan; 5 Department of Psychiatry, Chiayi Branch, Taichung Veterans General Hospital, Taipei, Taiwan; 6 Endoscopy Center of Diagnosis and Treatment, Taipei Veterans General Hospital, Taipei, Taiwan; 7 Division of Gastroenterology, Department of Medicine, Taipei Veterans General Hospital, Taipei, Taiwan; 8 Cancer Center, Taipei Veterans General Hospital, Taipei, Taiwan; 9 Department of Psychiatry, Taipei Veterans General Hospital, Taipei, Taiwan; 10 Department of Family Medicine, Taipei Veterans General Hospital, Taipei, Taiwan; 11 Department of Psychiatry, Su-Ao and Yuanshan Branch, Taipei Veterans General Hospital, Taipei, Taiwan; University of Michigan, United States of America

## Abstract

**Background:**

Studies have shown that chronic inflammation may play a vital role in the pathophysiology of both gastroesophageal reflux disease (GERD) and bipolar disorder. Among patients with GERD, the risk of bipolar disorder has not been well characterized.

**Objective:**

We explored the relationship between GERD and the subsequent development of bipolar disorder, and examined the risk factors for bipolar disorder in patients with GERD.

**Methods:**

We identified patients who were diagnosed with GERD in the Taiwan National Health Insurance Research Database. A comparison cohort without GERD was matched according to age, sex, and comorbidities. The occurrence of bipolar disorder was evaluated in both cohorts based on diagnosis and the prescription of medications.

**Results:**

The GERD cohort consisted of 21,674 patients, and the comparison cohort consisted of 21,674 matched control patients without GERD. The incidence of bipolar disorder (incidence rate ratio [IRR] 2.29, 95% confidence interval [CI] 1.58–3.36, *P*<.001) was higher among GERD patients than among comparison cohort. Multivariate, matched regression models showed that the female sex (hazard ratio [HR] 1.78, 95% CI 1.76–2.74, *P* = .008), being younger than 60 years old (HR 2.35, 95% CI 1.33–4.16, *P* = .003), and alcohol use disorder (HR 4.89, 95% CI 3.06–7.84, *P* = .004) were independent risk factors for the development of bipolar disorder among GERD patients.

**Conclusions:**

GERD may increase the risk of developing bipolar disorder. Based on our data, we suggest that attention should be focused on female patients younger than 60 years, and patients with alcohol use disorder, following a GERD diagnosis.

## Introduction

Gastroesophageal reflux disease (GERD) is a condition which develops when the reflux of stomach contents causes troublesome symptoms and/or complications. It is one of the most common gastrointestinal disorder which presents as heartburn and regurgitation. It significantly affects quality of life and healthcare costs and rapidly increases in Asian countries. [1]–[3] However, the mechanisms involved in the pathogenesis of GERD symptoms have not been fully elucidated. Studies have shown that, in GERD patients, the esophageal mucosa produces significantly more amounts of various cytokines including interleukin-6 (IL-6), IL-8, IL-1 beta, interferon gamma (IFN-gamma), tumor necrosis factor alpha (TNF-alpha) compared with healthy people. [4] These inflammatory mediators activate immune cell recruitment and migration, and may play an important role in the generation of GERD symptoms; in other words, GERD may be considered an inflammatory process. [4], [5].

In clinical care, interest in the psychiatric aspects of gastroenterological diseases has grown. [6] Increasing numbers of gastroenterological diseases, including GERD, have been proved to be associated with psychiatric disorders, in particular, depression, anxiety, and sleep disturbance. [7]–[10] Evidence has showed that reflux symptoms are more common in patients with bipolar disorder. [11] However, the relationship between GERD and bipolar disorder has been less studied.

Studies have shown that cytokines circulating in the plasma may impair the function of the blood-brain barrier, [12] which may indicate that peripheral inflammation is associated with the upregulation of central nervous system (CNS) inflammation. [13] Several studies have shown that chronic inflammation plays a critical role in the pathophysiology of common mental disorders, [14] including depression and bipolar disorder. [15], [16] Therefore, we hypothesized that a history of GERD increases the risk of the subsequent onset of bipolar disorder.

To prove our hypothesis, we designed a nationwide population-based study to investigate the incidence of bipolar disorder among patients with GERD.

## Patients and Methods

### Data Sources

The Taiwan’s National Health Insurance (NHI) program offers a comprehensive, unified, universal health insurance program to all residents of Taiwan. The NHI program covers more than 96% of Taiwan residents, and has contracted with 99% of the hospitals and clinics in Taiwan. [17] The program provides coverage for outpatient, inpatient, emergency, and traditional Chinese medicine services, as well as for prescription drugs. Multiple NHI databases, including NHI enrollment files, claims data, and a prescription drug registry, are managed and publicly released by the National Health Research Institutes (NHRI) of Taiwan. The Bureau of NHI and NHRI regulations guarantee patient confidentiality, and data identifying patients is encrypted. Detailed information about the data source is provided on the NHRI website (http://nhird.nhri.org.tw) and any problems about the data request could be sent to e-mail address at the NHRI (e-mail: nhird@nhri.org.tw).

### Ethics Statement

The Institutional Review Board of the Taipei Veterans General Hospital approved this study (2013-08-016BC). Written consent from study patients was not obtained because the NHI dataset consists of de-identified secondary data for research purposes, and the Institutional Review Board of Taipei Veterans General Hospital issued a formal written waiver for the need for consent.

### Study Design and Participants

We conducted a retrospective cohort study of patients newly diagnosed with GERD between April 1, 2000 and December 1, 2009. We identified GERD cases in the Taiwan National Health Insurance Research Database (NHIRD) based on the International Classification of Diseases, 9th revision, Clinical Modification (ICD-9-CM) codes 530.11 and 530.81. To increase the validity of GERD diagnoses, we included only the patients who received proton pump inhibitor (PPI) and diagnosed as GERD. The Bureau of NHI requires that patients with GERD be diagnosed by performing either endoscopy or 24-hour pH-meter monitoring before a PPI can be prescribed for treatment. Patients with bipolar disorder were identified based on diagnoses of mood or behavior disturbances, related to a principal diagnosis of a bipolar disorder (ICD-9-CM codes 296.0X, 296.1X, 296.4X, 296.5X, 296.6X, 296.7X, 296.80, or 296.89). We also analyzed the use of drugs approved by the FDA (Food and Drug Administration) for treating one (or more) phases of bipolar disorder; the drugs were classified according to the World Health Organization (WHO) Anatomical Therapeutic Chemical (ATC) classification. In our study, only patients who were prescribed these drugs for at least one month were included in our study. In addition, patients with mood disorders resulting from a general medical condition (ICD-9-CM code 293.83) and patients with a history of mood disorders before the enrollment date were excluded from our study.

For each patient with GERD in the NHIRD, a patient without GERD matched for age, sex, comorbidities, [18] and enrollment date was included in the comparison cohort. Although there are many studies which have found several comorbidities to be risk factors for GERD, based on our inflammation hypothesis in this study, other inflammation-related comorbidities may be considered as potential confounders. Identical exclusion criteria were applied to the matched comparison cohort. Both the GERD and comparison patients were followed until the development of bipolar disorder, death, or the end of 2010.

### Statistical Analysis

Diagnosis of bipolar disorder served as the primary dependent variable. We calculated bipolar disorder incidence rates (per 10,000 person-years) and incidence rate ratios (IRRs). The study groups were compared using the χ^2^ test for categorical variables. The Kaplan-Meier method was used to estimate the cumulative incidence of bipolar disorder, and a Cox proportional hazards model was used to identify risk factors for bipolar disorder in patients with GERD. The qualifying criterion for inclusion in the multivariate analysis was a result in the univariate-analysis with a *P* value less than 0.1. The Perl programming language (version 5.12.2) was used to extract the data from the databases. Microsoft SQL Server 2005 (Microsoft Corp., Redmond, WA, USA) was used to execute data linkage, processing, and control sampling. SPSS software, version 19.0 for Windows (IBM, Armonk, NY, USA), and SAS software, version 9.2 (SAS Institute, Cary, NC, USA) were used to perform all statistical analyses. Comparison results with *P* values less than.05 were considered statistically significant.

## Results

### Participant Characteristics

Table 1 shows the demographic and comorbidity data of the GERD patients and comparison participants. The median age of the patients was 52 years. The majority of patients in both cohorts were men (54.2%). Hypertension, dyslipidemia, and chronic obstructive pulmonary disease were the most common comorbidities. There were no statistically significant differences in the baseline comorbidity data between the study groups.

**Table 1:** Baseline characteristics of patients with gastroesophageal reflux disease (GERD) and matched cohort.

Demographic data	Patients with GERD		Matched cohort		*P* value
	(*n* = 21,674)		*n* = 21,674		
	*n*	%	*n*	%	
**Age (years) (interquartile range)**	52(40–65)		52(40–65)		
≥60	7,079	32.7	7,079	32.7	1.000
<60	14,595	67.3	14,595	67.3	
**Sex**					
Male	11,737	54.2	11,737	54.2	1.000
Female	9,937	45.8	9,937	45.8	
**Comorbidities**					
Alcohol use disorder	1,856	8.6	1,855	8.6	0.986
Autoimmune diseases	2,593	12.0	2,591	12.0	0.976
Chronic kidney disease	3,651	16.8	3,652	16.8	0.990
Cerebrovascular disease	4,333	20.0	4,331	20.0	0.981
Diabetes mellitus	5,974	27.6	5,976	27.6	0.983
Hypertension	9,289	42.9	9,289	42.9	1.000
Asthma	4,627	21.3	4,627	21.3	1.000
COPD	7,227	33.3	7,225	33.3	0.984
Malignancies	945	4.4	934	4.3	0.795
Cirrhosis	1,119	5.2	1,089	5.0	0.512
Dyslipidemia	8,444	39.0	8,443	39.0	0.992
Coronary artery disease	624	2.9	598	2.8	0.451
Obesity	483	2.2	469	2.2	0.646
**Follow-up years (median)**	3.03(1.86–4.39)		2.96(1.79–4.32)		<0.001

COPD, chronic obstructive pulmonary disease.

### Incidence of Bipolar Disorder

**Figure 1:**
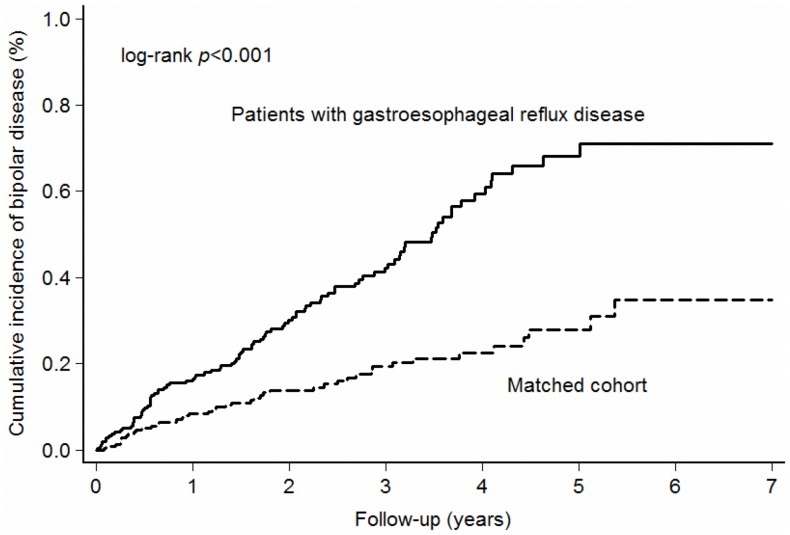
Cumulative incidence of bipolar disorder in patients with gastroesophageal reflux disease (GERD) and matched cohort.

The cumulative incidences of bipolar disorder are shown in Figure 1. As shown in Table 2, the risk of developing bipolar disorder was significantly higher for patients with GERD than for the matched control patients (IRR 2.29, 95% confidence interval [CI] 1.58–3.36, *P*<.001). After stratifying patients according to age and sex, we observed that patients with GERD aged less than 60 years were associated with a higher risk of developing bipolar disorder (IRR 2.59, 95% CI 1.70–4.03, *P*<.001), but patients aged more than 60 years were not. This enhanced risk was observed in both men and women. We also stratified patients according to follow-up duration, and observed that only patients with longer follow-up durations were associated with a higher risk of subsequent bipolar disorder (1–3 years and ≥3 years). Overall, our study showed that the incidence of the development of bipolar disorder after the diagnosis of GERD was 14.0 per 10,000 person-years.

**Table 2:** Incidence of bipolar disorder in patients with gastroesophageal reflux disease (GERD) and matched cohort.

	Patients with GERD		Matched cohort		IRR (95% CI)	*P* value
	Bipolar No.	Per 10,000person-year	Bipolar No.	Per 10,000person-year		
Total	96	14.0	43	6.1	2.29(1.58–3.36)	<0.001
Age						
≥60	14	6.7	11	5.0	1.34(0.57–3.27)	0.473
<60	82	17.3	32	6.7	2.59(1.70–4.03)	<0.001
Sex						
Male	44	11.8	20	5.2	2.27(1.31–4.06)	0.002
Female	52	16.8	23	7.3	2.30(1.38–3.94)	0.001
Follow-up						
0–0.5 year	20	18.7	11	10.2	1.83(0.84–4.23)	0.105
0.5–1 year	15	14.3	7	6.6	2.18(0.84–6.32)	0.086
1–3 year	40	13.0	17	5.4	2.41(1.34–4.54)	0.002
≥3 year	21	12.8	8	4.7	2.73(1.16–7.12)	0.012

IRR, incidence rate ratio; CI, confidence interval.

### Risks Factors for Bipolar Disorder in Patients with GERD

As demonstrated in the univariate and multivariate analyses, independent risk factors for the development of bipolar disorder among GERD patients were being younger than 60 years of age (HR 2.35, 95% CI 1.33–4.16, *P* = .003), female (HR 1.78, 95% CI 1.16–2.74, *P* = .008), and having an alcohol use disorder (HR 4.89, 95% CI 3.06–7.84, *P*<.001) (Table 3).

**Table 3:** Analyses of risk factors for bipolar disorder in patients with gastroesophageal reflux disease (GERD).

Predictive variables	Univariate analysis		Multivariable analysis	
	HR (95% CI)	*P* value	HR (95% CI)	*P* value
Age <60	2.58(1.46–4.54)	0.001	2.35(1.33–4.16)	0.003
Sex(female)	1.42(0.95–2.12)	0.090	1.78(1.16–2.74)	0.008
Comorbidities				
Alcohol use disorder	4.38(2.81–6.84)	<0.001	4.89(3.06–7.84)	<0.001
Autoimmune diseases	1.63(0.96–2.75)	0.069	1.59(0.93–2.71)	0.088
Chronic kidney disease	1.16(0.69–1.96)	0.579		
Cerebrovascular disease	0.64(0.35–1.16)	0.141		
Diabetes mellitus	1.33(0.87–2.04)	0.196		
Hypertension	1.12(0.75–1.67)	0.593		
Asthma	1.31(0.82–2.08)	0.256		
COPD	1.33(0.88–2.01)	0.170		
Malignancies	0.59(0.15–2.41)	0.465		
Cirrhosis	1.61(0.75–3.48)	0.225		
Dyslipidemia	1.26(0.84–1.89)	0.259		
Coronary artery disease	2.08(0.85–5.12)	0.111		
Obesity	2.41(0.98–5.92)	0.056	1.96(0.79–4.86)	0.144

COPD, chronic obstructive pulmonary disease.

## Discussion

This is the first population-based study to examine GERD as a risk factor for bipolar disorder by using a matched cohort design and a long-term follow-up period. This study observed a higher incidence of the development of subsequent bipolar disorder among patients with GERD. In addition, GERD patients who were female and aged less than 60 years had a greater risk of developing bipolar disorder than those who were male and aged more than 60 years. Alcohol use disorder was another risk factor for the development of bipolar disorder among patients with GERD.

In our study, patients with GERD were determined to be at higher risk for developing subsequent bipolar disorder. We hypothesize that this may be attributed to two possible mechanisms. First, the development of bipolar disorder after the onset of GERD may be the result of an inflammatory process activated by GERD. In patients with GERD, the esophageal mucosa produces higher amounts of various cytokines including IL-6, IL-8, IL-1 beta, IFN-gamma, TNF-alpha. [4] Even in non-erosive reflux disease (NERD), which the role of inflammation may be considered less obvious, enhanced expression of IL-8 and IL-1 beta has been found. [19], [20] The chronic peripheral inflammatory process activated by GERD may increase the risk of subsequent bipolar disorder by upregulating CNS inflammation. [13] Studies have revealed that chronic, mild inflammation in the periphery and in the brain occurs in bipolar disorder. [21], [22] Cytokines have been shown to access the brain and interact with pathophysiological domains relevant to bipolar disorder. Using animal models, it is shown that peripheral cytokines reach the brain through various mechanisms, including a leaky brain barrier, active transport, the activation of endothelial cells, and binding to cytokine receptors. [23] Levels of proinflammatory cytokines such as IL-2, IL-4, and IL-6 are elevated during mania, whereas IL-6 is elevated during depression. [24] Second, GERD and bipolar disorder share common risk factors, such as stress. Laboratory stress has been found to increase the perception of intraluminal acid stimuli and induced stress, anxiety, anger in GERD patients rather than normal control. [25] Stressful psychosocial factors can induce GERD, [26] and stress may also induce bipolar disorder in patients genetically prone to developing bipolar disorder. [27], [28] Psychological stress also may activate inflammatory responses in the brain. [29].

When stratifying according to follow-up duration, the risk of bipolar disorder among GERD patients was significantly higher after the first year following the GERD diagnosis, which is consistent with our hypothesis that inflammation is responsible for the association between GERD and bipolar disorder. We hypothesize that long periods of time are required for the chronic inflammatory process. Based on our results, detection bias was unlikely.

In our study, we observed that younger age was an independent risk factor for developing subsequent bipolar disorder among GERD patients. The incidence of bipolar disorder is determined to be relatively rare in people aged more than 60 years. [30] Our study confirmed this finding.

Epidemiological studies have shown that bipolar disorder is equally prevalent among men and women. However, in this study, we observed that women with GERD had a greater risk of developing a bipolar disorder than men did. One possible explanation is that women in our study group, with a median age of 52 years, were vulnerable to fluctuating estrogen levels, thereby increasing their risk of developing bipolar disorder. [31], [32].

In our analysis of the risk factors associated with subsequent bipolar disorder in GERD patients, alcohol use disorder was an independent risk. Evidence has shown that alcohol use disorder and bipolar disorder share certain common genetic characteristics, neuroimaging findings, and biochemical findings. [33], [34].

This is the first retrospective study to examine GERD as a risk factor for the development of bipolar disorder. This study’s strengths were its matched case-control design using a population-based cohort of GERD patients, and adequate controls for comorbidity. However, several limitations inherent to the use of claims databases must be considered. First, the results of endoscopies and patient’s symptoms could not be obtained from the database. Consequently, the influence of GERD severity as a risk factor for developing subsequent bipolar disorder could not be determined. Second, the causal relationship between GERD and bipolar disorder was assessed mainly by determining the time of onset of these two conditions in particular patients. However, both conditions may need a long periods to seek treatment; thus, the possibility that bipolar disorder caused GERD cannot be completely ruled out. Finally, many demographic variables were unavailable in the database, such as socioeconomic status, lifestyle, and family medical history; analysis of these variables may have provided useful information regarding additional factors associated with GERD and bipolar disorder. [35]–[38].

In conclusion, the results of this study suggested that GERD increases the risk of developing bipolar disorder. Based on our data, we suggest that attention should be focused on female patients, patients aged less than 60 years, and patients with alcohol use disorders, following GERD diagnosis. Further prospective clinical studies on the relationship between GERD and bipolar disorder are warranted.
